# Therapeutic Potential and Cellular Mechanisms of Panax Notoginseng on Prevention of Aging and Cell Senescence-Associated Diseases

**DOI:** 10.14336/AD.2017.0724

**Published:** 2017-12-01

**Authors:** Haiping Zhao, Ziping Han, Guangwen Li, Sijia Zhang, Yumin Luo

**Affiliations:** ^1^Cerebrovascular Diseases Research Institute, Xuanwu Hospital of Capital Medical University, Beijing, China; ^2^Center of Stroke, Beijing Institute for Brain Disorders, Beijing, China; ^3^Beijing Key Laboratory of Translational Medicine for Cerebrovascular Diseases, Beijing, China

**Keywords:** Panax notoginseng, aging, neurodegenerative disease, vessel, cancer

## Abstract

Owing to a dramatic increase in average life expectancy, most countries in the world are rapidly entering an aging society. Therefore, extending health span with pharmacological agents targeting aging-related pathological changes, are now in the spotlight of gerosciences. *Panax notoginseng* (Burk.) F. H. Chen, a species of the genus Panax, has been called the "Miracle Root for the Preservation of Life," and has long been used as a Chinese herb with magical medicinal value. *Panax notoginseng* has been extensively employed in China to treat microcirculatory disturbances, inflammation, trauma, internal and external bleeding due to injury, and as a tonic. In recent years, with the deepening of the research pharmacologically, many new functions have been discovered. This review will introduce its pharmacological function on lifespan extension, anti-vascular aging, anti-brain aging, and anti-cancer properties, aiming to lay the ground for fully elucidating the potential mechanisms of *Panax notoginseng’s* anti-aging effect to promote its clinical application.

## 1. Introduction

With a dramatic increase in life expectancy, most countries are entering an aging society. How to delay aging and to prevent cell senescence-associated diseases has become a hotspot of research all over the world. As a consequence, pharmacological agents targeting aging-related pathological changes are now in the spotlight of geroscience [1]. In recent years, hormone replacement using estrogen and testosterone has become a focus of “anti-aging” medicine. However, negative consequences might happen when indiscriminately targeting senescent cells for anti-aging therapy [2]. Traditional Chinese medicine (TCM) is a holistic, natural health care system that offers a different perspective characterized by the nourishing of life and, its role in anti-aging is getting more and more attention.

*Panax notoginseng* (Burk.) F. H. Chen is a species of the genus Panax, which is called “sānqī” in Chinese. Three major species most extensively researched and used worldwide for thousands of years as either food or medicine are, *Panax notoginseng* (Burk.) F.H. Chen (notoginseng), *Panax ginseng C.A. Meyer* (Asian ginseng) and *Panax quinquefolius* (American ginseng) [3]. *Panax notoginseng,* called the "miracle root for the preservation of life," has long been used as a Chinese herb with magical medicinal value.

Aging is the result of both physiological and pathological processes that lead to a progressive functional decline in cells, tissues, and organisms. Several major theories including the mitochondrial free radical theory [4], deregulated metabolic and immune responses [5], genetic and epigenetic regulation of aging [6], telomere shortening theory [7], and stem cell theory [8] are thought to play a significant role in aging.

Chronic inflammation, lipid deposition, oxidative stress, reduced cell proliferation, irreversible growth arrest and cell apoptosis are the characteristics of vascular aging [9]. Macrophage uptake of oxidized LDL results in the formation of foam cells, fatty streaks and eventually fibrous plaques [10]. Therefore, lowering the serum cholesterol and antioxidant therapy could prevent vascular aging and reduce the incidence of cardiovascular and cerebrovascular diseases [11]. *Panax notoginseng* has been used for the treatment of microcirculatory disturbances, inflammation, and internal and external bleeding due to injury.

Brain aging leads to different degrees of behavioral and cognitive dysfunction. In aging humans, the most common neurodegenerative disorder is Alzheimer’s disease (AD) and Parkinson's disease (PD), which results in mitochondrial dysfunction, oxidative stress, inflammation, and cytotoxic substance accumulation. Aging-induced immune senescence occurs in the brain as microglia senescence, which functions abnormally and promotes neurodegeneration [12, 13]. *Panax notoginseng* contains many active ingredients and is shown to have a role of anti-brain aging.

Cancer is an age-related disease, especially cancer of the breast, lung, prostate, and colon. About 60% of cancer diagnoses occur in the 13% of the population aged 65 years or older, who have a decline in immune competence, less resistance and longer exposure to carcinogens, decreased DNA repair, defects in tumor-suppressor genes. Recently, the anti-tumor effect of *Panax Notoginseng* has been revealed. Dietary and medicinal uses of *Panax notoginseng* have been associated with reduced risk of cancer.

Substantial efforts have been made to research the phytochemistry and pharmacological effects of *Panax notoginseng*, which led to the isolation of over 200 compounds and discovery of a variety of pharmacological effects. This review will introduce its pharmacological function on lifespan extension, anti-vascular aging, anti-brain aging and anti-cancer effects, aiming to lay the ground for fully elucidating the potential mechanisms of *Panax notoginseng’s* anti-aging properties to promote its clinical application.

## 2. Ethnopharmacology, phytochemistry, and pharmacology of *Panax notoginseng*

*Panax notoginseng*, distributed in the Southwest of China, Japan, Burma, and Nepal, is both cultivated and gathered from wild forests. *Panax notoginseng* is sensitive to sunlight. It grows primarily in the Wenshan mountain area of Yunnan province and is cultivated commercially in the Southwest regions of China. Currently, *Panax notoginseng* is a unique botanical plant since its cultivation strictly follows the GAP guidelines [14]. It has been widely used for over 400 years and still holds a unique position in today’s regional market, with remarkable annual sales of 5000 tons in China alone.

### 1.1 Ethnopharmacology of *Panax notoginseng*

*Panax notoginseng*, classified in Chinese medicine, is warm, sweet, slightly bitter, and non-toxic. Unlike many other herbal medicines with a highly variable range of applications, *Panax notoginseng* has always been used for more focused purposes. Traditional pharmacopeia recommended it among the most effective herbs for promoting blood circulation and hemostasis. The herbalogical study shows that *Panax notoginseng* originated from the “Compendium of Materia Medica,” stating: "Sanqi is a herb belonging to the blood phase of the yang ming and jue yin meridians, it can treat all diseases of the blood." Eventually, this drug was used to remove stasis, reduce bleeding and swelling, alleviate pain, mainly used in surgery and traumatology. For example, "Golden mirror of medicine" uses it to remove necrotic tissue and to promote granulation. "Records of Traditional Chinese and Western Medicine in Combination" uses it to treat hemoptysis, hematemesis, hematochezia, hematuria and other various types of bleeding. "A Supplement to the Compendium of Materia Medica" uses it to treat traumatic hemorrhage. *Panax notoginseng* is the main ingredient in Yunnan Bai Yao, a popular hemostatic proprietary herbal remedy to deal with wounds, stop bleeding, decrease inflammation and relieve pain [15].

### 1.2 Phytochemistry of *Panax notoginseng*

Phytochemical studies, on the root, stem, leaf, and flower of *Panax notoginseng, have been conducted*. As recently reviewed by Wang [16], over 200 chemical constituents were isolated from *Panax notoginseng*, including saponins, polysaccharides, dencichine, amino acids, flavonoids, phytosterols, cyclopeptides, saccharides, fatty acids, volatile oils, aliphatic acetylene hydrocarbons, and trace elements. Saponins are the major constituents of *Panax notoginseng* and considered as the primary active compounds. More than100 saponins have been isolated and identified, including ginsenosides, notoginsenosides, and gypenosides. Flavonoids isolated from *Panax Notoginseng* are mainly flavonols and flavone glycosides, such as liquiritigenin, quercetin, and kaempferol-3-O-α-L-rhamnoside. Recently, only 14 cyclopeptides were isolated, and they were all cyclodipeptides. Sterols isolated from *Panax notoginseng* include β-sitosterol, daucosterol, stigmasterol, stigmasterol-3-O-β-D-glucopyranoside, and stigmast-7-en-3β-ol-3-O-β-D-glucopyranoside. Reports regarding polyacetylenes in *Panax notoginseng* are rare. Saccharides in *Panax notoginseng* include monosaccharides, oligosaccharides, and polysaccharides. There are more than 19 kinds of amino acids in *Panax notoginseng*, and 7 of them are considered essential amino acids. The volatile constituents of *Panax notoginseng* include terpenes, alcohols, aldehydes, olefins, and alkanes. Terpenes are a significant component because of its relatively high percentage among these compounds. Although there are a lot of active components in *Panax notoginseng*, the current pharmacological studies mainly focus on the constituents of saponins.

### 1.3 Pharmacology of *Panax notoginseng*

*Panax notoginseng*, a valuable traditional Chinese medical herb, has numerous pharmacological effects. Protective actions of *Panax notoginseng* against cardiovascular diseases and diabetes have been reviewed [17-20], along with its pharmacological actions including hemostatic and wound healing activity, antioxidant activity, anti-inflammatory activity, hypoglycemic and anti-hyperlipidemic activities, anti-coagulation activity, neuroprotective effects, hepatoprotective effects, renoprotective effects, anti-tumour activity, and estrogen-like activities. A bulk of pharmacological studies were focused on the saponins or steryl glycosides, though polysaccharides with immunopotentiating properties, proteins with antifungal, ribonuclease and xylanase activity, and a triacylglycerol (trilinolein) with antioxidant activity have been reported [21]. These might contribute to the mechanism of *Panax notoginseng* on anti-aging and aging-associated diseases.

## 3. Anti-aging and anti-aging related effects of extractions of *Panax notoginseng* (EPN)

Chemical characteristics are diverse in different parts including rhizome, root, fiber root, seed, stem, leaf, and flower of *Panax notoginseng*. Therefore, the pharmacological function of various parts is also different.

### 3.1 Anti-vascular aging by EPN

EPN has therapeutic effects through multiple pharmacological actions including antioxidation, lipid-lowering, and prevention of vascular remodeling.

#### Antioxidant function

In vitro study showed that EPN delays Ang II-induced aging of human umbilical vein endothelial cells (HUVECs) by downregulating the expression of NADPH oxidase subunit-p47phox through angiotensin II type I receptor (AT1R), and further reducing the reactive oxygen species (ROS) production [22].

#### Lipid-lowering effect

Lipid-lowering medications have been shown to reduce both atherogenic lipoproteins [23]. *Panax notoginseng*, on a dietary supplement-treated SD rats on a high-fat diet, showed a significant decline in serum levels of total cholesterol, triglycerides, and LDL-cholesterol, with an increase in serum HDL-cholesterol levels, accompanied by a reduced level of hepatic HMG-CoA reductase. *Panax notoginseng* also improves hepatic antioxidant status as assessed by superoxide dismutase and glutathione peroxidase activities and reduced levels of lipid peroxidation [24]. These results suggest that *Panax notoginseng* intake can improve lipid profiles, restrain peroxidation, and enhance the activity of antioxidant enzymes. These features are likely beneficial for reducing the risk of coronary heart disease associated with hyperlipidemia and oxidative stress.

#### Prevention of vascular remodeling

Aging and hypertension could result in an excessive proliferation of rat aortic vascular smooth muscle cells (VSMCs) and the expression changes of correlated cytoactive factors. EPN can lower their proliferation levels and reduce the expressions of negative cytokines, thus reducing aging and hypertension induced injury of VSMCs and delaying angiocellular aging. EPN can slow vascular aging of SHR rats, which works by p16-cyclin D/CDK-RB pathway to inhibit VSMC proliferation [25]. Remodeling of Adventitia was seen in aged rats, which was manifested as thickened adventitia and accumulated collagens with disordered ratios of collagen I and III. Its mechanism might be possibly associated with activation of the renin-angiotensin system (RAS). EPN could improve adventitial remodeling of 20-month senescent rats possibly by interfering multi-targets, such as Ang II and AT1R, thereby delaying vascular aging. Taken together, *Panax notoginseng* also has direct inhibition on vascular remodeling. The anti-vascular aging function and mechanism of extracts and bioactive components of *Panax notoginseng* was summarized in table 1.

**Table 1:** Anti-vascular aging function and mechanism of extracts and bioactive components of *Panax notoginseng*.

Compounds	Cell/tissue	Effects	Mechanisms	Refs.
Extracts from P. notoginseng	HUVECs	Anti-oxidation	angiotensin II type I receptor↓ expression of NADPH oxidase subunit-p47phox↓ superoxide anion production↓	[22]
	rats	Lipid-lowering effect	serum levels of total cholesterol triglycerides, and LDL-cholesterol↓ serum HDL-cholesterol levels↑ hepatic HMG-CoA reductase↓	[24]
	VSMCs	Inhibit VSMCs proliferation	p16-cyclin D/CDK-RB pathways↓	[25]
	rats	Inhibition of adventitia remodeling	Ang II levels in adventitia↓ expression of AT1R↓ type III/I collagen area ratio↑	[25]
PNS	HCAECs	Anti-inflammation	TNF-α-induced monocyte adhesion↓ ICAM-1 and VCAM-1 in vitro and in vivo↓	[36]
	HUVECs	Anti-inflammation	ox-LDL-induced monocyte adhesion↓ ICAM↓	[37]
	zymosan A treated rats	Anti-inflammation	inflammatory factors, such as integrins, IL-18, IL-1β and MMP-2, and MMP-9↓ NF-κB expression↑ IκB-α expression↑	[37]
	rabbits	Anti-inflammation	mRNA levels of MCP-1, IL-6, C-reactive protein and NF-κB in the aorta wall↓	[38]
	ApoE knockout mice	Anti-oxidation	plaque area↓ serum levels of lipid and oxLDL↓ expression of CD40 and MMP-9↓	[39]
	zymosan A treated rats	Anti-inflammation	phosphorylation of FAK↓ integrins expression↓ NF-κB translocation↓	[40]
	rats	Lipid-lowering effect	transcriptional activation of the LXRα gene promoter↑ ABCA1 and ABCG1↑ NF-κB DNA binding activity↓	[41]
	rats	Inhibition of intimal hyperplasia	expression of PCNA↓ cyclin E, cyclin D1, fibronect, and MMP-9↓	[42]
	VSMCs	Inhibit VSMCs proliferation	cell cycle-related factors and ERK signal transduction↓	[44]
	VSMCs	Inhibit VSMCs proliferation	p53, Bax, and caspase-3 expressions↑ Bcl-2 expression↓	[45]
	HUVECs	antiangiogenesis	VEGF-KDR/Flk-1and PI3K-Akt-eNOS signaling pathways	[46]
Ginsenoside Rg1	VSMCs	D-galactose-induced senescence	p16INK4a/Rb and p53-p21Cip1/Waf1 signaling pathways↓	[58]
Notoginsenoside R1	HUVECs HPAECs		modulate the fibrinolytic capacity expression of tPA and decreasing PAI-1 activity↑	[99] [100]
	HASMCs		TNF-a-induced PAI-1 overexpression↓ ERK1/2 and PI3K/Akt signaling pathways↑	[101]
	human endothelial EA. hy926 cells	Anti-inflammation	oxLDL-induced inflammatory cytokines production↓ PPARγ↑ oxLDL-induced NF-κB and MAPK activation↓	[102]
	ApoE knockout mice	Anti-oxidation	serum levels of GSH and SOD↑ level of MDH↓	[103]
	HUVECs	promotes angiogenesis	HIF-1a-mediated VEGF expression↑ PI3K/AKT and Raf/MEK/ERK signaling↑	[104]

**Table 2:** Anti-tumor effect and mechanism of extracts and bioactive components of *Panax notoginseng*.

Compounds	Cells/tissues	Effects	Mechanisms	Refs.
Extracts from P. notoginseng	human colorectal carcinoma SW480 cells	Anti-colorectal cancer	arrest the cells in S and G2/M phases.	[27]
	human colorectal carcinoma SW480 cells	Anti-colorectal cancer	arrested cells in the synthesis phase cyclin A expression↑	[28]
	SW480 human colorectal cancer cells	Chemotherapy sensitizer	enhanced the actions of 5-fluorouracil and irinotecan	[28]
	hepatoma Hep3B cells, Hep3B implanted SCID mice	antiproliferation activity	reduced tumor volume and weight	[29]
PNS	human colon cancer LoVo cell	Anti-colorectal cancer	cell cycle arrest at S phase antioxidative capacities	[52]
	4T1 cells	Anti-breast carcinoma	genes known to inhibit metastasis↑ genes promoting metastasis in cultured↓	[53]
	HeLa cells	Chemotherapy sensitizer	cisplatin cytotoxicity↑ gap junctions activit	[54]
	Lewis lung carcinoma cells C56BL/6J male	Anti-tumor accompanied by cardiovascular disorders	expression of CD34 and vWF in tumor↓ expression of vascular markers in heart↑	[55]
Ginsenoside Rg1	HeLa cells	Chemotherapy sensitizer	cisplatin cytotoxicity↑ gap junction activity↑	[72]
Ginsenoside Rd	AGS and MCF-7 cells	inhibit cell proliferation of gastric and breast cancer	TRPM7 channel activity↓	[86]
	HeLa cell	inhibits proliferation and induces apoptosis	Bcl-2 expression↓Bax expression↑ mitochondrial transmembrane potential↓ activating the caspase-3 pathway↑	[87]
	hepatocellular carcinoma HepG2 cell	inhibit metastasis	MAPK signaling↓ focal adhesion formation↑	[88]
	mammary carcinoma 4T1 cells	attenuate breast cancer metastasis	depressing miR-18a-mediated Smad2 expression regulation	[89]
Ginsenoside Re	AGS cells	inhibits proliferation	p21 level↑ phosphorylation of CDK2↓ S phase arrest↑ caspase-8, caspase-9, and caspase-3↑	[98]
Notoginsenoside R1	HCT-116 cells	Inhibition of metastasis	integrin-1 protein↓, E-selectin, ICAM-1) ↓	[111]
	HeLa cells	Chemotherapy sensitizer	enhanced cisplatin cytotoxicity enhancement of gap junction’s activity	[72]
	BALB/c mice	Anti-lung carcinogenesis	lung cancer stem cells↓ epithelial-tomesenchymal transition↓	[113]
Notoginsenoside Ft1	SH-SY5Y cells	Anti-neuroblastoma	arrested the cell cycle at S, G2/M stages cell apoptosis↑ p38 MAPK and ERK1/2 pathways↑	[112]
Polysaccharide	murine H22 hepatocarcinoma	Anti-hepatocarcinoma	activated CD4(+) T-cells↑ serum IL-2↑	[115]
Trilinolein	non-small cell lung carcinoma A549	inhibits proliferation	modulating PI3K/Akt pathway	[123]

### 3.2 Anti-neurodegeneration by EPN

Immune senescence induced by aging manifests in the brain as age-associated microglia senescence. EPN suppressed microglial activation as measured by reduced expression of accessory molecules (CD40 and CD86), decreased production of inflammatory mediators (IL-6 and TNF-α), and diminished release of antibacterial products (nitric oxide). This immunosuppressive activity was neither dependent on the glucocorticoid receptor, nor the result of a single ginsenoside (Rb1, Rg1, or Re), which are the major active constituents of the whole extract [26].

### 3.3 Anti-tumor by EPN

EPN from four extracts including root, rhizome, flower, and berry can arrest the human colorectal cancer SW480 cells in S and G2/M phases, and induce apoptosis. The flower extracts had stronger anti-proliferative effects on SW480 cells when compared with the other three extracts [27]. The EPN from root arrested cells in the S phase and increased cyclin A expression [28]. Moreover, *Panax notoginseng* fermentation broth showed anti-proliferation activity against hepatoma Hep3B cells, indicated by reduced tumor volume and weight in treated Hep3B implanted SCID mice [29]. Apart from these, EPN could enhance the actions of 5-fluorouracil and irinotecan on SW480 cells, suggesting that *Panax notoginseng* can reduce the needed dose of chemotherapeutic agents to achieve desired effects. Together, these results indicate that EPN may provide significant natural defense against human cancer via inhibiting cell proliferation and promoting cell apoptosis by regulating distinct molecules. Further studies in vivo and human trials are needed to confirm its efficacy and safety. The anti-tumor effect and mechanism of extracts and bioactive components of *Panax notoginseng* was summarized in table 2.

**Figure 1.**
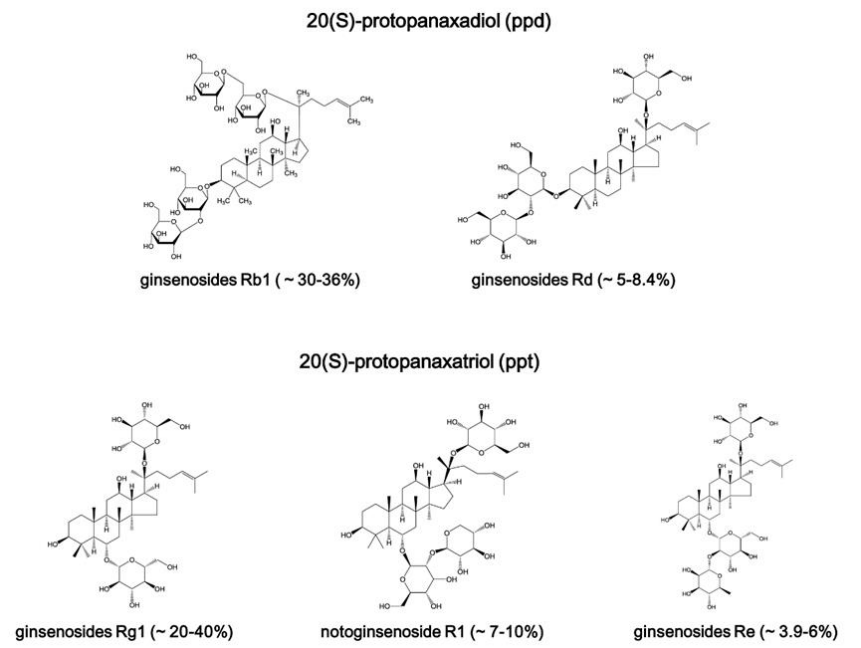
Chemical structure and proportion of five main compounds of *Panax notoginseng* saponins.

## 4. Anti-aging activity of bioactive components of *Panax notoginseng*

### 4.1 Anti-aging related effects of total *Panax notoginseng* saponins (PNS)

Saponins are the main active ingredients of *Panax notoginseng*, and more than 100 saponins have been identified. PNS belongs to dammarane-type ginsenoside, which includes two classifications: the 20(S)-protopanaxadiol (PDS) and 20(S)-protopanaxatriol (PTS). PNS contains high levels of ginsenoside Rb1, Rd (PDS classification) and ginsenoside Rg1, Re, notoginsenoside R1 (PTS classification). The top five saponins ginsenoside Rb1, ginsenoside Rg1, notoginsenoside R1, ginsenoside Rd and ginsenoside Re, constitute up to 90% of total PNS used in pharmacological experiments (Fig. 1).

#### 4.1.1 Antioxidant function of PNS

D-galactose could induce senescence model in vitro and in vivo. PNS reduced D-galactose-induced apoptosis of rat cardiomyoblasts H9c2 cell line through upregulation of antioxidative ability [30]. This provides a scientific basis for further exploitation of the mechanism of longer life span controlled by *Panax notoginseng*.

#### 4.1.2 Immunoregulatory function of PNS

Aging is associated with multiple alterations in the proliferative and functional properties of the immune system. These are not linked to any pathology but has consequences in immune-senescence and inflammation. Aging-associated changes in B-cell function can be investigated at a mechanistic level. For examples, reduced expression of genes important for lineage commitment and differentiation results in diminished B-cell production. PNS, PDS, ginsenosides-Rb1, -Rd, notoginsenosides-K,-R4 from the root could enhance specific antibody and the activation potential of both T and B cells against ovalbumin in mice [31-34]. T helper 17 (Th17) cells have been implicated in the development of autoimmune and chronic inflammatory diseases in humans. Recent studies have indicated that PNS functions as an anti-inflammatory agent, which was demonstrated by controlling the proliferation and differentiation of Th17 cells through global downregulation of the expression of inflammatory cytokines and cell cycle genes [35].

#### 4.1.3 Anti-vascular aging by PNS

##### Anti-inflammatory function

The initial stage of atherosclerosis development is indicated by monocyte adhesion to the endothelium. Among the saponin fractions (PNS, PDS, and PTS), PDS is the most potent fraction against TNFα-induced monocyte adhesion and the expression of adhesion molecules ICAM-1 and VCAM-1 in vitro and in vivo [36]. Another study showed that PNS reverses ox-LDL-induced HUVECs injuries by decreasing the expression of ICAM-1 and the adhesion rate with monocytes. PNS exerts its therapeutic effects on atherosclerosis of rats on a high-fat diet by reducing the expression of inflammatory factors including integrins, IL-18, IL-1β, possibly through attenuating the expression of NF-κB and increasing IκB-α expression [37]. PNS attenuates atherogenesis in the aorta wall of rabbits through decreasing the levels of MCP-1, IL-6, C-reactive protein and NF-κB, as well as the blood lipid profile [38]. PNS can also lower the plaque area in ApoE knockout mice, reduced serum levels of lipid, and oxLDL, downregulate the expression of CD40 and MMP-9 [39]. PNS inhibits zymosan A-induced atherogenesis in rats by suppressing phosphorylation of FAK, integrins expression, and NF-κB translocation [40].

##### Lipid-lowering effect

PNS treatment alleviated the typical pathological changes associated with atherosclerosis in rats. PNS-mediated attenuation of AS may, at least partly, be due to LXRα upregulation, which is demonstrated by enhanced transcriptional activation of the LXRα gene promoter and subsequent upregulation of ABCA1 and ABCG1 and inhibition of NF-κB DNA binding activity [41].

##### Prevention of vascular remodeling

Several lines of evidence demonstrate that PNS could inhibit the vascular intimal hyperplasia and the VSMCs proliferation. In vivo, PNS from root inhibit vessel restenosis in rats after vascular intimal injury, indicated by the blockage of the VSMCs proliferation, and the reduction of ECM protein deposition in the endometrium. This effect may be related to the downregulated expression of proliferating cell nuclear antigen, cyclin E, cyclin D1, fibronectin, and MMP-9 [42]. In vitro, PNS can inhibit VSMCs proliferation stimulated by hypercholesterolemic serum and hyperlipidemia serum [43]. The impact was through inhibiting the activation of ERK pathway [44], upregulating p53, Bax, and caspase-3 expressions and downregulating Bcl-2 expression [45]. Besides, PNS can promote angiogenesis, and that the proangiogenic effects involve the VEGF-KDR/Flk-1 and PI3K-Akt-eNOS signaling pathways [46].

#### 4.1.4 Anti-neurodegeneration by PNS

##### Antioxidant function

Oxidative stress due to accumulation of ROS is involved in cell death associated with neurological disorders such as stroke, AD, PD and traumatic brain injury. The protective effects of PNS from leaves on H_2_O_2_-induced cell death in primary rat cortical astrocytes were associated with attenuation of ROS accumulation, which involved activation of Nrf2 and upregulation of downstream antioxidant systems [47]. PTS, an inducer of Trx-1, has pluripharmacological properties in the protection against PD including enhancing antioxidant activity, acting as a neurotrophic factor, modulating inflammation and inhibiting mitochondria-mediated apoptosis [48].

##### Reduction of cytotoxic substance accumulation

The amyloid hypothesis states that problems with increased levels of beta-amyloid cause Alzheimer's disease. Three proteases, α-secretase, β-secretase and γ-secretase are involved in the processing of amyloid precursor protein [49]. PNS can improve the abilities of learning and memory of senescence accelerated mouse-prone 8 (SAMP8) mice, which may be relevant to down-regulating the expression of APP gene at the transcriptional level. PNS modulates the level of protein and gene expressions involved with α and β secretase, thereby increasing α-secretase activity and reducing β-secretase activity, which may be one of the mechanisms of PNS precluding Aβ generation [50]. Phosphorylated and truncated tau has been documented during the progression of AD as well as their capacity to exert cytotoxicity when expressed in cells and animal models. PNS upregulated the synaptophysin gene expression at the transcriptional level in the brain of SAMP8 mice but did not significantly affect the expression of Tau gene. Accordingly, PNS may be a promising agent for Alzheimer's disease.

##### Reduction of intracellular calcium overload and neurotransmitter balance

Acetylcholine (ACh) is a vital neurotransmitter in the brain, the abnormal activity of choline acetyltransferase (ChAT) and acetylcholinesterase (AChE) can cause Ach metabolic disorders, leading to biochemical changes in the central cholinergic nervous system. PNS plays a protective role against the loss of cholinergic neurons in an AD rat model typified by reductions in the level of ChAT and the number of cholinergic neurons.

##### Promotion of neuroregeneration

PNS can increase the number of nestin-, proliferating cell nuclear antigen-, Tuj-1-, neurofilament-, vimentin-, glial fibrillary acidic protein-, bFGF-, and BDNF-positive cells from cortical stem cells isolated from rat embryos on embryonic day 17. This increase implies that PNS can promote rat embryonic cortical NSC survival, self-renewal, proliferation, and differentiation through neurotrophic factors by autocrine or paracrine signaling [51].

#### 4.1.5 Anti-tumor by PNS

PNS could inhibit the proliferation and metastasis of the cancer cells. PNS was found to have a markedly cytotoxic effect and antiproliferative activity against the human colon cancer cell line LoVo in a dose- and time-dependent manner arresting cell cycle at S phase [52]. PNS halted the human colorectal cancer SW480 cells in the S phase and significantly increased cyclin A and cell apoptosis expression [28]. PNS inhibited highly metastatic breast carcinoma cell line 4T1 migration and invasion [53]. Moreover, PNS significantly enhanced cisplatin cytotoxicity through enhancement of GJ activity in a dose-dependent manner in HeLa cells [54].

Tumor, when associated with cardiovascular disorders, creates a greater challenge for clinical management given the paradoxical involvement of angiogenesis. PNS and its predominant active components Rg1, Rb1 and R1 suppressed tumor growth and simultaneously attenuated myocardial ischemia. PNS treatment led to decreased expression of miR-18a, CD34, and vWF in tumor and increased expression of miR-18a and vascular markers in the heart of mice implanted with Lewis lung carcinoma cells. The tissue specific regulatory effects on angiogenesis in part through modulating the expression of miR-18a, which could be responsible for its bi-directional effect on angiogenesis [55]. These studies provide experimental evidence warranting evaluation of PNS and related bioactive component as a rational therapy for complex disease conditions including co-manifestation of cancer and ischemic cardiovascular disease.

### 4.2 Anti-aging and anti-aging related effects of Ginsenoside Rg1

#### 4.2.1 Immunoregulatory effects of Ginsenoside Rg1

It is a well-documented fact that aging leads to a substantial decline in T cell function. The possible reasons for the decline include the inability of lymphocytes to proliferation in response to mitogenic stimulation and the decrease of IL-2 production. Ginsenoside Rg1 given in vivo and in vitro enhanced the proliferation of lymphocytes and the production of IL-2 in aged rats [56]. However, it had no influence on the immune function in young and adult rats. Thus, it is reasonable to consider Rg1 as an “immunoregulator” rather than an “immunopotentiating agent Further investigation suggested that the mechanism underlying Rg1’s effect on immune function in aged rats might be involved in increasing cAMP and cGMP levels in lymphocytes [57].

#### 4.2.2 Anti-vascular aging by Ginsenoside Rg1

Ginsenoside Rg1 can inhibit D-galactose-induced VSMCs senescence, and the mechanisms may be related to its partial inhibition of the p16INK4a/Rb and p53-p21Cip1/Waf1 signaling pathways during the cell cycle [58].

#### 4.2.3 Anti-neurodegeneration by Ginsenoside Rg1

A systematic review revealed that Ginsenoside Rg1 had the greatest effect on acquisition and retention memory in AD models [59]. Rg1 has a protective effect on dopaminergic neurons in the substantia nigra striatum of 6-OHDA-induced rat PD model, which was related to the insulin like growth factor receptor signaling pathway [60]. Rg1 treatment succeeded in restoring motor functions to physiological level in MPTP-induced mouse PD model, which was accompanied by an attenuation of the MPTP-induced loss of dopaminergic neurons in the SN and striatum [61]. In addition, Rg1 could attenuate LPS-induced inflammatory responses via the phospholipase C-gamma1 signaling pathway in murine BV2 microglial cells [62].

##### Regulation of cytotoxic substances accumulation

Ginsenoside Rg1 induces neuroprotection through ameliorating amyloid pathology, improving cognition, and activating PKA/CREB signaling in transgenic APP mice [63, 64]. Rg1-treatment increased ADAM10 level while reduced BACE1 level and apoptosis on an ovariectomized and D-galactose-injected rat model of AD, which supports the potential application of Rg1 in the treatment of learning and memory impairments in postmenopausal women [65]. Moreover, Rg1 can also activate peroxisome proliferator-activated receptor to upregulate the IDE expression to enhance the Aβ degradation in a rat model of AD [66]. Rg1 has estrogen activity, which can activate the MAPK/ERK signaling pathway in human platelets to regulate the metabolism of APP and prevent the occurrence of AD [67]. Rg1 protects against Aβ-induced neuronal apoptosis via estrogen receptor alpha and glucocorticoid receptor-dependent anti-protein nitration pathway [68]. Additionally, Rg1 also antagonizes Aβ25-35 induced endothelial cell apoptosis by reducing HIF-1α-activated protein tyrosine nitration and inhibition of mitochondrial apoptotic cascade [69].

##### Regulation of neurotransmitter balance

Ginsenoside Rg1 considerably enhanced the learning and memory dysfunction caused by beta-AP(25-35), and this beneficial effect could be attributed to its inhibition of AchE and enhancement of ChAT activity [70]. Rg1 and Rb1 can increase the Ach level in the hippocampus, but Rg1 inhibited AchE activity, while Rb1 did not affect the activity of AchE. Rg1 and Rb1 can inhibit the reduction of 5-hydroxytryptamine induced by scopolamine serotonin, indicating that Rg1 and Rb1 both are effective in improving memory, but probably through different mechanisms [71].

#### 4.2.4 Anti-tumor effect of Ginsenoside Rg1

Ginsenoside Rg1 significantly enhances cisplatin cytotoxicity in HeLa cells with functional GJs, which leads to a considerable enhancement of a dye-coupled GJ in a dose-dependent manner [72]. These results indicate that Rg1 is the active compound enhancing the cytotoxic action of cisplatin induced by PNS in the presence of functional GJs.

### 4.3 Anti-aging and anti-aging related effects of Ginsenoside Rb1

#### 4.3.1 Anti-stress effects of Ginsenoside Rb1

Various kinds of stress, especially chronic stress, cause many diseases and accelerate aging. Treatment with Rb1 before repeated hanging stress could prevent the decrease of sexual behavior and the increase of corticorsterone, and brought the plasma concentration of sexual hormones back to normal level [73]. Further study proved that Rb1 significantly improved sexual function, and the underlying mechanism might be the activation of NO/cGMP pathway in mice corpus cavernosum [74]. Based on these results, Rb1 is believed to be the main anti-stress principle in *Panax notoginseng,* and it might be a promising candidate for prevention and treatment of stress-related diseases.

#### 4.3.2 Anti-neurodegeneration by Ginsenoside Rb1

Ginsenoside Rb1 prevents MPP-induced apoptosis in PC12 cells by stimulating estrogen receptors with sequential activation of ERK1/2, Akt, and inhibition of SAPK/JNK, p38 MAPK [75]. Rb1 protected PC12 cells against Aβ-induced injury through inhibition of ROS production and an increase in Bcl-2/Bax and inhibition of caspase-3 activity [76]. PI3K/Akt/GSK-3β pathway was implicated in Rb1's attenuation of beta-amyloid-induced neurotoxicity [77]. Besides, Rb1 could selectively block L-type voltage-gated calcium channel, thereby inhibiting the Aβ25-35-induced voltage-gated calcium channel currents without influence on Aβ25-35-induced an intracellular calcium release in hippocampal neurons [78]. Moreover, pretreatment with Rb1 suppressed the protein expression of phosphorylated Tau and upregulated the expression levels of BDNF in brain slice of okadaic acid-induced AD model [79]. Post treatment of Rb1 improved the learning and memory and reduced the tau phosphorylation by reversing the p-GSK3 and PP2A level [80]. In addition, Rb1 attenuated the activation of LPS-induced brain microglia activation in animals [81].

### 4.4 Anti-aging and anti-aging related effects of Ginsenoside Rd

#### 4.4.1 Antioxidant function of Ginsenoside Rd

Ginsenoside Rd attenuates the oxidative damage of SAM mice at 11 months of age, which may be responsible for the intervention of GSH/GSSG redox status [82]. In addition, Rd potentiated H_2_O_2_-induced apoptosis of basilar artery smooth muscle cells through the mitochondria-dependent pathway [83].

#### 4.4.2 Immunoregulatory effects of Ginsenoside Rd

Ginsenoside Rd significantly enhanced the Con A-, LPS-, and OVA-induced splenocyte proliferation in the OVA-immunized mice. Meanwhile, the production of the Th1 and Th2 cytokines were significantly enhanced by Rd. Further, Rd significantly increased the IL-2, interferon-gamma, IL-4, and IL-10 mRNA expression in mice splenocyte induced by Con A. These data imply that Rd has immunological adjuvant properties, and elicits a Th1 and Th2 immune response by regulating the production and gene expression of Th1 cytokines and Th2 cytokines[84].

#### 4.4.3 Promotion of neuroregeneration by Ginsenoside Rd

PNS and Ginsenoside Rd promote the differentiation of neurospheres into astrocytes. Rd increases the production of astrocytes in a dose-dependent manner. On the other hand, both PNS and Rd induce a weak but significant effect by decreasing the number of neurons [85].

#### 4.4.4 Anti-tumor effect of Ginsenoside Rd

Rd could inhibit tumor cell proliferation and metastasis. It was found that Rd could inhibit gastric and breast cancer cell proliferation and survival in vitro by inhibiting type transient receptor potential 7 (TRPM7) channel activity [86], and inhibits HeLa cell proliferation and induces cell apoptosis by down-regulating Bcl-2 expression, enhancing Bax expression, reducing the mitochondrial transmembrane potential, and activating the caspase-3 pathway [87]. Moreover, Rd has been shown to inhibit hepatocellular carcinoma HepG2 cell metastasis via inactivation of MAPK signaling and induction of focal adhesion formation [88], and attenuate breast cancer metastasis in mouse mammary carcinoma 4T1 cells in part through depressing miR-18a-mediated Smad2 expression regulation [89].

### 4.5 Anti-aging and anti-aging related effects of Ginsenoside Re

#### 4.5.1 Antioxidant function of Ginsenoside Re

Ginsenoside Re protects cardiomyocytes from oxidant injury induced by both exogenous and endogenous oxidants, which may be predominantly attributed to scavenging H_2_O_2_ and hydroxyl radicals [90]. Re could be a potential anti-oxidant to protect HUVECs against oxidative stress damage. Proteomic analysis showed that the expression of 23 protein spots was upregulated in Re and H_2_O_2_ groups to resist oxidative stress [91]. These studies might offer novel nsights into the mechanisms of Re in protecting the cardiovascular system.

#### 4.5.2 Immunoregulatory effects of Ginsenoside Re

Re enhanced viability of CD4+ T cells through the regulation of IFN-gamma-dependent autophagy activity [92].

#### 4.5.3 Anti-neurodegeneration by Ginsenoside Re

Re-increased the expression of cholinergic markers and neuronal differentiation in Neuro-2a cells, which may counter the symptoms and progress of AD [93]. Also, Re prevented PC 12 cells from lesion induced by serum-free medium and beta-amyloid peptide [94]. Re inhibits BACE1 through activation of PPARγ, which ultimately reduces the generation of Aβ [95]. Therefore, Re may be a promising agent for the modulation of Aβ-related pathology in AD. Re demonstrated protection of nigral neurons from MPTP-induced apoptosis in a mouse model of PD, and this effect is likely attributable to its ability to enhance the expression of Bcl-2 proteins, downregulate the expression of Bax and iNOS protein, and inhibit the activation of caspase-3 [96]. Re was neuroprotective against LPS-treated BV2 microglial cells via the phospho-p38, iNOS, and COX2 signaling pathways, suggesting that Re exerts a beneficial effect on neuroinflammatory events in neurodegenerative diseases [97].

#### 4.5.4 Anti-tumor effect of Ginsenoside Re

The products of heat-processed Re inhibited phosphorylation of CDK2 at Thr160 by upregulation of p21 level, resulting in S phase arrest. The products of heat-processed Re also activated caspase-8, caspase-9, and caspase-3, followed by cleavage of PARP, in a dose-dependent manner. The anticancer effects of the products of heat-processed Re in AGS cells are largely facilitated via generation of less-polar ginsenosides Rg6 and F4 [98].

### 4.6 Anti-aging and anti-aging related effects of Notoginsenosides

Notoginsenoside R1 (NR1) is a phytoestrogen, which is found only in PNS and has a history of prevention and treatment of cardiovascular diseases. Notoginsenoside Ft1 (Ft1), another important saponin isolated from *Panax notoginseng*, was used not only as a P2Y12 agonist in enhancing platelet aggregation, but also as a stimulator of proliferation, migration, and tube formation in cultured HUVECs.

#### 4.6.1 Anti-vascular aging by Notoginsenoside

NR1 purified from *Panax notoginseng* can modulate the fibrinolytic capacity of endothelial cells in vitro by increasing the expression of tPA and decreasing PAI-1 activity [99, 100]. Further study demonstrated that NR1 inhibited TNF-a-induced PAI-1 overexpression via ERK1/2 and PI3K/Akt signaling pathways [101]. NR1 could suppress oxLDL-induced inflammatory cytokines production via activating PPARγ, subsequently inhibiting oxLDL-induced NF-κB and MAPK activation [102]. In vivo study showed that NR1 significantly alleviated the atherosclerotic lesion in ApoE2/2 mice marked by a reduction in lipid deposition, fibrosis and oxidative stress, accompanied by increased serum levels of GSH and SOD and decreased levels of MDH, reduced levels of inflammatory [103]. Ft1 promotes angiogenesis in cultured HUVECs via HIF-1a mediated VEGF secretion and the regulation of PI3K/AKT and Raf/MEK/ERK signaling pathways in HUVECs [104].

#### 4.6.2 Anti-neurodegeneration by Notoginsenoside

##### Antioxidant function

Notoginsenosides can reduce ROS and confer neuroprotective effects. Preincubation with NR1 significantly counteracted the effects of Aβ by increasing cell viability, reducing oxidative damage, restoring mitochondrial membrane potential, and suppressing stress-activated MAPK signaling pathways in a cell-based model of AD [105]. Notoginsenoside R2 showed neuroprotective effects against 6-Hydroxydopamine-induced oxidative stress and apoptosis in SH-SY5Y cells, which mediated P90RSK and Nrf2 activation via MEK1/2-ERK1/2 pathways [106].

##### Regulation of cytotoxic substances accumulation

NR1 inhibited Aβ accumulation and increased insulin degrading enzyme expression in both APP/PS1 double-transgenic mice and N2a-APP695sw cells, suggesting that NR1 may exert its protective effects through the enhancement of Aβ degradation and the effect of NR1 was partly mediated by PPARγ [107].

##### Regulation of neurotransmitter balance

Oral administration of NR1 improved the learning performance of the APP/PS1 mouse model of AD. In addition, NR1 reversed Aβ1-42 oligomers-induced impairments in long term potentiation. NR1 increased the membrane excitability of CA1 pyramidal neurons in hippocampal slices by lowering the spike threshold possibly through a mechanism involving the inhibition of voltage-gated K^(+)^ currents [108]. Imbalance in the regulation of NMDA receptor activity may be the basis for many central nervous system diseases, such as AD, PD and ischemic brain damage [109]. NR1 can selectively act on the NR1/NR2B subtype of NMDA receptor to inhibit the intracellular calcium overload induced by glutamate, which can protect the neurons in mice [110].

#### 4.6.3 Anti-tumor by Notoginsenoside

NR1 affects human colorectal cancer HCT-116 cells metastasis by inhibiting cell migration, invasion, and adhesion and by regulating expression of metastasis-associated signaling molecules [111]. Moreover, NR1 significantly enhanced cisplatin cytotoxicity in HeLa cells with functional GJs and led to significant enhancement of a dye-coupled GJ in a dose-dependent manner [72]. Among the saponins examined, Ft1 showed the best inhibitory effect on cell proliferation of SH-SY5Y cells, which not only arrested the cell cycle at S, G2/M stages but also promoted cell apoptosis. It might function possibly via p38 MAPK and ERK1/2 pathways, which indicates the potential therapeutic effect of it on human neuroblastoma [112]. Although individual treatment with three natural compounds (NR1, shikonin, and aconitine) may prevent cancer, they were not effective for the treatment of established tumors. However, combination treatment almost entirely stopped urethane-induced lung carcinogenesis and reduced tumor burden, demonstrated by reduced number of lung cancer stem cells by inducing cell differentiation, restoration of gap junction intercellular communication and blockade of the epithelial to mesenchymal transition [113].

**Figure 2.**
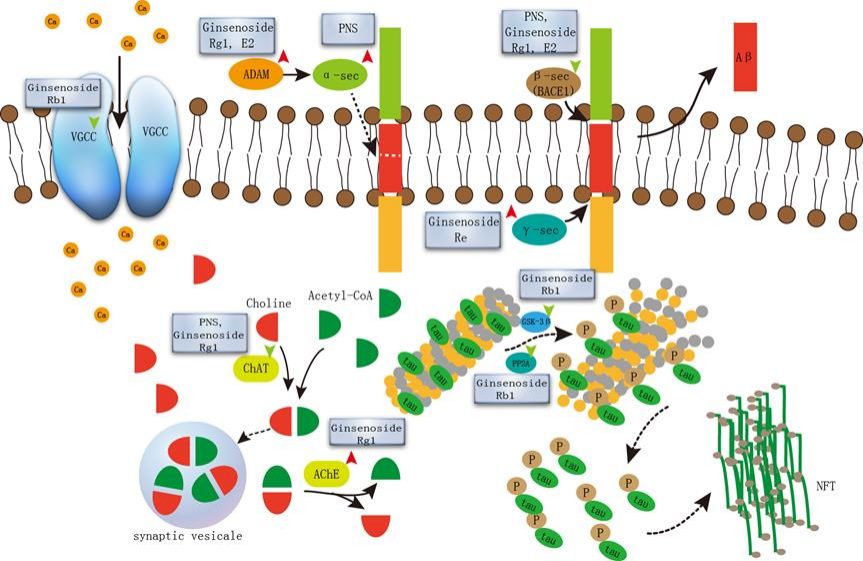
Illustration of the cellular and molecular targets of total and individual *Panax notoginseng* saponins (PNS) on neural cells destroyed by Alzheimer's disease (1) Prevention of Aβ formation and Amelioration of Aβ cytotoxicity. In the non-amyloidogenic pathway, α-secretase (α-sec) cleaves amyloid precursor protein (APP) within the Aβ domain, therefore precludes the formation of Aβ. In the amyloidogenic pathway, β-secretase (β-sec) and γ-secretase cleaves APP to produce Aβ. (2). Reduction of intracellular calcium overload. Increased calcium entered cells via the voltage-gated calcium channel currents (VGCC), resulting in calcium overload. (3) Regulation of Tau protein phosphorylation. In AD, there is a reduction in the ability of Tau to bind to tubulin and promote microtubule assembly. Hyperphosphorylated Tau contributes to the destabilization of microtubules and ultimately the formation of neurofibrillary tangle (NFT). (4) Increasing the activation of the cholinergic nervous system. Acetylcholine (ACh) is synthesized in the cytosol of cholinergic presynaptic neurons from choline and acetyl-coenzyme A (acetyl-CoA) by the enzyme choline acetyltransferase (ChAT) and is then transferred into synaptic vesicles. In the synaptic cleft, ACh is rapidly hydrolyzed by the enzyme acetylcholinesterase (AChE), releasing acetate and choline. Red arrow, promotion; green arrow, inhibition.

### 4.7 Anti-aging and anti-aging related effects of polysaccharides of *Panax notoginseng* (PPN)

The PPN has attracted more attention due to its immunomodulating activities.

#### 4.7.1 Lifespan extension by PPN

The PPN might be considered as a potential source to delay aging. It was demonstrated that PPN (main root polysaccharide, branch root polysaccharide, and fibrous root polysaccharide) especially the main root polysaccharide significantly extended the lifespan of C. elegans. Polysaccharides had little scavenging ability of ROS in vitro, while the heat stress resistance effect of polysaccharides on C. elegans might be attributed to the elevation of antioxidant enzyme activities and the reduction lipid peroxidation of malondialdehyde level [114]. Administration of PPN prolonged the survival of H22 tumor-bearing mice, and the increase in activated CD4+ T cells and the elevation of serum IL-2 may contribute to the antitumor activity [115]. These results provided a scientific basis for the further exploitation of the mechanism of longer lifespan controlled by *Panax notoginseng*.

**Figure 3.**
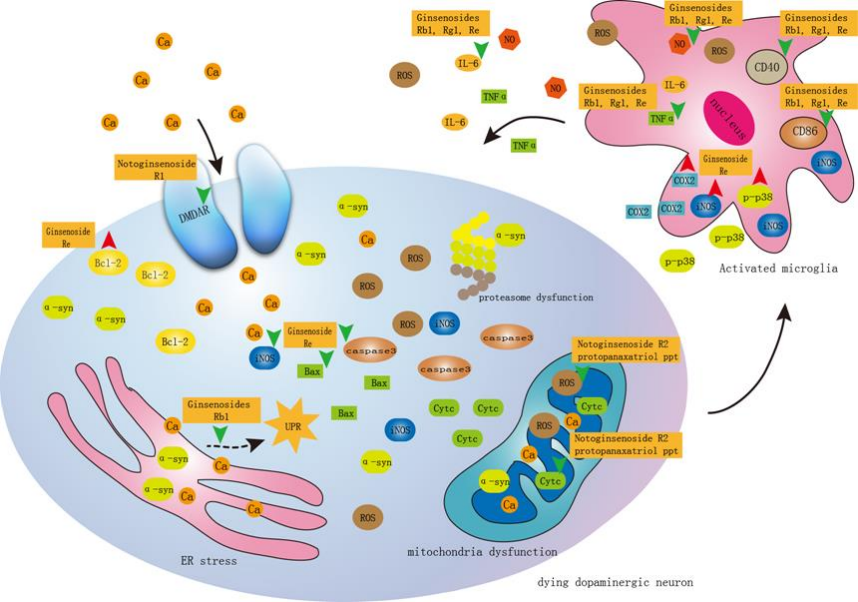
Pharmacological effects and mechanisms of *Panax notoginseng* saponins (PNS) against Parkinson's disease (1) Protection of dopaminergic neurons through inhibition of oxidative stress and ER stress. (2) Acting on NMDA receptors. Notoginsenoside R1 can selectively act on the NR1/NR2B subtype of NMDA receptor to inhibit the intracellular calcium overload induced by glutamate, which protect the neurons in mice. (3) Regulation of microglia activation. PNS suppressed microglial activation through depression of accessory molecules (CD40 and CD86), decreased production of inflammatory mediators (IL-6 and TNFα), and diminished release of antibacterial products (nitric oxide).

#### 4.7.2 Immunoregulatory effects of PPN

The total polysaccharide fraction has been demonstrated to stimulate the proliferation of murine spleen lymphocytes in vivo as well as in vitro, and to oppose the action of the T-cell suppressor, cyclosporin A[116]. The water-soluble high molecular weight fraction caused the production of IFN-γ and IFN-α in mouse spleen lymphocytes and peritoneal macrophage cell cultures. On the other hand, the weak alkali-soluble fraction showed anti-complement activity, as well as cytokine induction activity [117]. A fraction, with a molecular weight of 1,500kDa, isolated from the roots PPN, showed the ability to activate the reticuloendothelial system [118].

### 4.8 Anti-aging and anti-aging related effects of trilinolein

Trilinolein is a triacylglycerol, with the fatty acid, linoleic acid, which carries two unsaturated bonds (C 18:2), at all three esterified positions of glycerol. Trilinolein has been shown to have various beneficial effects, including reducing thrombogenicity, erythrocyte deformability, and arrhythmias and having antioxidant effects in different experimental models [17]

#### 4.8.1 Antioxidant function by trilinolein

Trilinolein quenched free radical-generated luminol chemiluminescence following the addition of phorbol myristic acetate in medium containing leukocytes. Trilinolein showed concentration dependent antioxidant activity [119, 120]. Incubation with trilinolein for 2 day increased both the activity and mRNA levels of SOD in rat aortic smooth muscle cells. However, after 7 day incubation with trilinolein, both the activity and mRNA levels of SOD were lowered in a dose-dependent manner, emphasizing the importance of choosing an optimal dosage for supplementation with antioxidants for scavenging oxygen free radicals [121]. The antioxidant capacity of trilinolein has also been demonstrated in brain astrocytes, liver and spleen [121, 122].

#### 4.8.2 Anti-tumor effect of trilinolein

Trilinolein inhibits proliferation of human non-small cell lung carcinoma A549 by modulating PI3K/Akt pathway [123].

## 5. Conclusing remarks

In recent years, *Panax notoginseng* has become one of the most popular health care products, especially for many older adults who eat little every day and have hypertension, hyperlipidemia, hyperglycemia, and other medical ailments. This review focuses on the significant role of the main components of *Panax notoginseng* on prevention of aging and cell senescence-associated diseases. Firstly, extractions and bioactive components of *Panax notoginseng* might be considered as a potential source to extend lifespan. Secondly, extractions and bioactive components of *Panax notoginseng* slow the vascular aging through lipid-lowering effect, anti-inflammation, antioxidation, inhibition of smooth muscle cell proliferation, inhibition of adventitia remodeling. Thirdly, mechanism of extractions and bioactive components of *Panax notoginseng* on neurodegenerative diseases were summarized. And, its monomer components for specific targets on AD and PD have become a hot research topic. The notogisenoside such as the NR1, Rg1, and Rb1 have been shown to have estrogen like activity [65, 68]. Many studies have demonstrated that estrogen has a role in neural protection [124]; however, the side effects of estrogen cannot be ignored[124]. Therefore, notogisenoside has little side effects of the plant estrogen, has a larger space for development in the treatment of aging related neurodegenerative diseases to replace hormone therapy (Fig. 2 and 3). Fourthly, extractions and bioactive components of *Panax notoginseng* have a broad range of anticancer activities in colorectal cancer, hepatocarcinoma, breast carcinoma, neuroblastoma, lung carcinogenesis, as well as tumor accompanied by cardiovascular disorders, which also enhanced the cytotoxicity of chemotherapeutic agents.

Although studies on the pharmacological effects of *Panax notoginseng* on aging-related diseases were numerous, most of the literature is aimed at the PNS monomer composition (Rg1, Rb1, F1), and some on the study of the *Panax notoginseng* saponins (PNS, PTS, PDS). However, studies on compatibility application of monomer composition with other traditional Chinese medicine were rare and needs further investigation in the future. It should be noted that when NR1 co-administration is done with drugs that are metabolized by CYP1A2, the possible herb-drug interactions should be monitored [125]. In addition, based on the theory of Traditional Chinese medicine, people without blood stasis, and people with blood deficiency are not suitable for taking this medication. Therefore, it is advisable to consult with a well-trained doctor of traditional Chinese medicine to discuss an individual plan that employs ancient Chinese wisdom.
